# EEG Phase-Amplitude Coupling Strength and Phase Preference: Association with Age over the First Three Years after Birth

**DOI:** 10.1523/ENEURO.0264-20.2021

**Published:** 2021-06-17

**Authors:** Michael G. Mariscal, April R. Levin, Laurel J. Gabard-Durnam, Wanze Xie, Helen Tager-Flusberg, Charles A. Nelson

**Affiliations:** 1 Department of Neurology, Boston Children’s Hospital, Harvard Medical School, Boston, MA 02115; 2 Division of Developmental Medicine, Boston Children’s Hospital, Harvard Medical School, Boston, MA 02215; 3 Department of Psychological and Brain Sciences, Boston University, Boston, MA 02215; 4 Harvard Graduate School of Education, Cambridge, MA 02138

**Keywords:** connectivity, cross frequency, development, EEG, phase-amplitude coupling, phase preference

## Abstract

Phase-amplitude coupling (PAC), the coupling of the phase of slower electrophysiological oscillations with the amplitude of faster oscillations, is thought to facilitate dynamic integration of neural activity in the brain. Although the brain undergoes dramatic change and development during the first few years of life, how PAC changes through this developmental period has not been extensively studied. Here, we examined PAC through electroencephalography (EEG) data collected during an awake, eyes-open EEG collection paradigm in 98 children between the ages of three months and three years. We employed non-parametric clustering methods to identify areas of significant PAC across a range of frequency pairs and electrode locations, and examined how PAC strength and phase preference develops in these areas. We found that PAC, primarily between the α-β and γ frequencies, was positively correlated with age from early infancy to early childhood (*p* = 2.035 × 10^−6^). Additionally, we found γ over anterior electrodes coupled with the rising phase of the α-β waveform, while γ over posterior electrodes coupled with the falling phase of the α-β waveform; this regionalized phase preference became more prominent with age. This opposing trend may reflect each region’s specialization toward feedback or feedforward processing, respectively, suggesting opportunities for back translation in future studies.

## Significance Statement

The brain undergoes significant development during infancy and early childhood, enabling the emergence of higher-level cognition. Phase-amplitude coupling (PAC) is thought to support the integration of information within the brain. Our data suggest PAC increases from three months to three years of age. We additionally report the anterior and posterior electrodes show opposing forms of PAC; this regional phase preference also increases with age of electroencephalography (EEG) collection. These findings help set the stage for future analyses of PAC in young children with neurodevelopmental disorders, in which development of biomarkers during early life is a burgeoning field and measures of cross-frequency coupling may offer new promise.

## Introduction

Dynamic integration of neural activity across various timescales is a critical component of healthy brain function. Oscillatory brain activity, created by the synchronous firing of large ensembles of neurons, can stem from a variety of underlying mechanisms and index a variety of cognitive processes (Kopell et al., 2014). In the field of neurodevelopment, measurement of oscillatory activity within specific frequency bands has thus been of longstanding interest (Saby and Marshall, 2012). Cross-frequency coupling, which describes the interaction between different oscillatory frequencies, has gained interest recently given its potential to integrate neural information in a manner that is functionally relevant to both typical and atypical brain function (Canolty and Knight, 2010).

One form of cross-frequency coupling is phase-amplitude coupling (PAC), in which the phase of the low-frequency activity modulates the amplitude of the high-frequency activity. Increasing evidence implicates PAC in a variety of important functional processes. PAC strength and location has been shown to shift with task demands (Voytek, 2010). Additionally, alterations in PAC have been found in a variety of brain disorders, including schizophrenia, autism, attention deficit hyperactivity disorder, and Parkinson’s (Salimpour and Anderson, 2019), highlighting the importance of PAC for healthy brain functioning.

Moreover, underlying characteristics of PAC may reflect an area’s functional configuration within neural networks. PAC phase max, or the phase of the low-frequency waveform corresponding to the largest amplitude of the high-frequency waveform, has been found to differ by cortical layer (Sotero et al., 2015), interneuron function (Port et al., 2019), depth of anesthesia (Soplata et al., 2017), brain area (Ninomiya et al., 2015), coupling frequencies, and task performance (Jones et al., 2020; Lega et al., 2016). This suggests that PAC phase preference (as measured on the scalp) could reflect an underlying area’s dynamic functional configuration during the recording period. Although many measures of PAC are agnostic to phase, these findings suggest that not just if, but how γ interacts with lower frequency waveforms may reflect a region’s role in either the intake or modulation of information for neural processing.

Numerous developmental changes in electroencephalography (EEG) power have been documented (Saby and Marshall, 2012). However, no studies have examined the development of PAC phase preference, and few have examined the development of PAC strength over early childhood. Of note, coupling between high δ (2–4 Hz) and a 40-Hz frequency following response during a 40-Hz auditory click train has been found to increase from 8 to 16 years in the first principal component of the EEG data back-projected onto FCz, and begins to decrease from 16 to 22 years (Cho et al., 2015). Still, few studies have focused on infancy and early childhood, a period when the brain is highly plastic and shows significant structural and functional developmental changes. Although PAC has been documented in infancy (Vanhatalo et al., 2005), only one study examined PAC development during infancy: PAC between slow wave (0.7–2 Hz) and β (11.3–32 Hz) oscillations across the scalp during sleep was found to decrease over the first two weeks after birth (Tokariev et al., 2016). These studies suggest that PAC can vary based on the age, frequency range, topography, state, and task being studied.

Thus, although the brain continues to demonstrate high plasticity and significant developmental changes from birth to early childhood (Gao et al., 2017), no studies have examined how PAC strength or phase preference develops in this age range. This is of particular importance because many neurodevelopmental disorders first manifest behaviorally during these early years. Development of predictive or risk-associated biomarkers that may allow for earlier treatment is a high priority in the field of neurodevelopmental disorders (Sahin et al., 2019; Wolff and Piven, 2020). While EEG power has been extensively studied for this purpose (Brito et al., 2019; Gabard-Durnam et al., 2019), PAC may offer particular promise given its potential mechanistic underpinnings (Canolty and Knight, 2010; Jensen et al., 2014; Hyafil et al., 2015; Helfrich et al., 2016). However, use of PAC for studying neurodevelopmental disorders first requires an understanding of how PAC changes across typical development. Here, we sought to address these gaps by characterizing, through both PAC strength and phase preference, how PAC develops between three months and three years of age.

## Materials and Methods

### Participants

The data for this study were drawn from a larger longitudinal study of neurocognitive development across the first three postnatal years of life. This study included later born infants (*n* = 98; 53 males and 45 females; Table 1) who had at least one typically developing older sibling. The study was conducted at Boston Children’s Hospital/Harvard Medical School and Boston University. All infants had a minimum gestational age of 36 weeks, no history of prenatal or postnatal medical or neurologic problems, no known genetic disorders (e.g., fragile X, tuberous sclerosis) and no family history of autism spectrum disorder or other neuropsychiatric conditions (based on parent report). Institutional review board approval was obtained from both institutions (#X06-08-0374).

**Table 1:** Participant demographics

	Participants (*n* = 98)
Sex	53 (M) 45 (F)
Maternal education	
<4-year degree	13 (13%)
4-year degree	26 (27%)
Graduate degree	49 (50%)
Did not answer	10 (10%)
Paternal education	
<4-year degree	19 (19%)
4-year degree	34 (35%)
Graduate degree	34 (35%)
Did not answer	11 (11%)
Race	
White or White	82 (84%)
Black or African American	3 (3%)
Asian	2 (2%)
American Indian or Alaskan Native	0 (0%)
Native Hawaiian or Pacific Islander	0 (0%)
More than one reported	10 (10%)
Not reported	1 (1%)
Ethnicity	
Not Hispanic or Latino	93 (94%)
Hispanic or Latino	4 (4%)
Not reported	1 (1%)

### EEG acquisition/processing

Among infants enrolled in the study, EEG data were collected sequentially at 3, 6, 9, 12, 18, 24, and 36 months of age, as previously described (Gabard-Durnam et al., 2019). However, not all participants completed an EEG recording at every time point; 17 participants had an EEG completed at only one time point, 10 participants had an EEG completed at two time points, 21 participants had three time points, 22 participants had four, 19 participants had five, eight participants had six, and only one participant had an EEG completed at all seven timepoints. Infants were seated on their caregiver’s lap while a research assistant blew bubbles and/or presented toys to ensure the infant remained calm, without any time-locked stimulus or task. Continuous EEG was recorded for up to 5 min using either a 64-channel Geodesic Sensor Net System or 128-channel Hydrocel Geodesic Sensor Nets (Electrical Geodesics). Data were sampled at either 250 or 500 Hz, and referenced at collection to a single vertex electrode (Cz). Impedances were kept below 100 kΩ (within recommended guidelines for young children, given the high-input impedance capabilities of this system’s amplifier).

**Figure 1.**
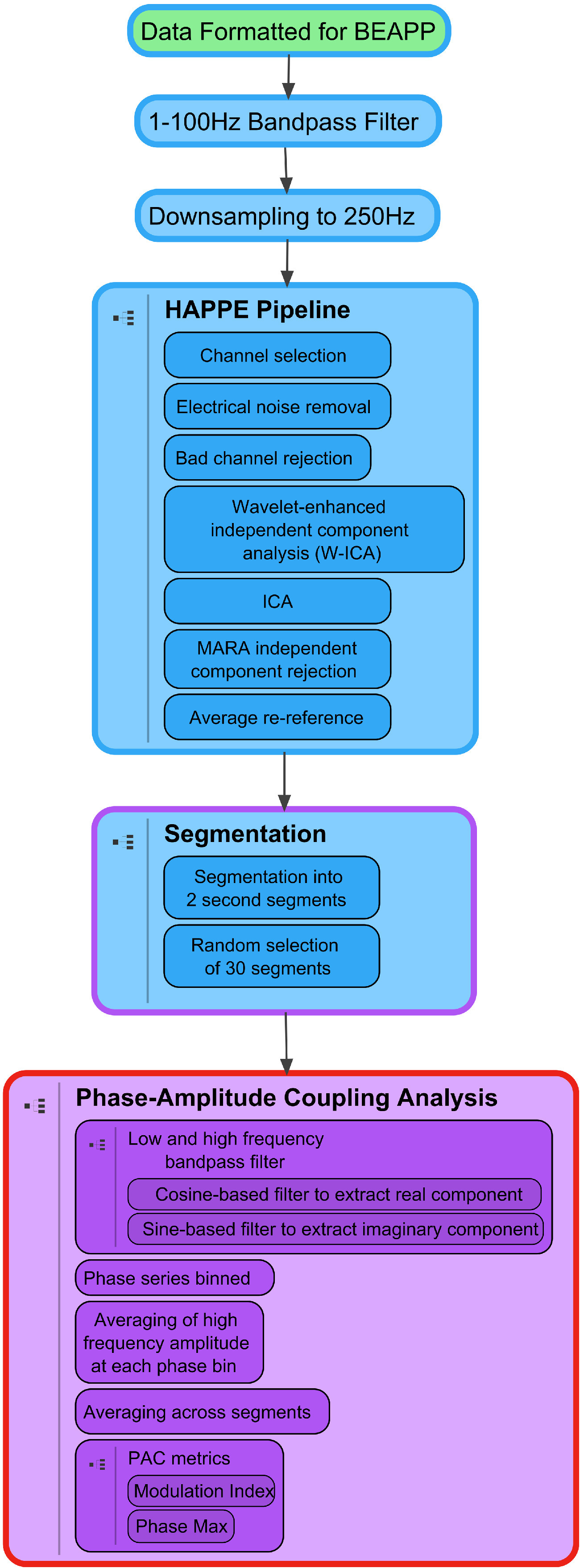
Processing pipeline: steps in EEG processing and PAC analysis. EEG data processing in BEAPP consists of several major steps: preprocessing (formatting, filtering, downsampling, HAPPE artifact rejection and re-referencing to average, segmentation) and PAC analysis; each step is represented by a cell, with additional details inside each cell. Cell body color reflects module input format, and cell outline color reflects module output format. Green = native file format. Blue = EEG in continuous array. Purple = EEG in segmented 3D array. Red = PAC measures.

Raw EEG files were exported from NetStation to MATLAB (versionR2017b, MathWorks) for preprocessing. Files were processed using the batch EEG automated processing platform (BEAPP; Levin et al., 2018; Fig. 1). Data were first filtered using a 1–100 Hz bandpass filter, which had a combined transition band width of 1 Hz. Data sampled at 500 Hz were then downsampled to 250 Hz for consistency, and to constrain the signal decomposed by later independent components analysis (ICA)-based steps (Gabard-Durnam et al., 2018). Subsequently, within BEAPP, the Harvard Automated Preprocessing Pipeline for EEG (HAPPE), which was developed specifically to optimize preprocessing of developmental EEG data with potentially high levels of artifact and short recordings, was used to automate preprocessing and artifact minimization (Gabard-Durnam et al., 2018). Because ICA cannot reliably decompose data with a large number of channels given a short recording (Onton and Makeig, 2006; Gabard-Durnam et al., 2018), and because many other electrodes differed in their placements between the 64-channel and 128-channel nets, only electrodes in the international 10–20 system were included in this pipeline; all other electrodes were removed before further analysis. In brief, within HAPPE, data underwent the following steps: electrical line noise removal at 60 Hz via Cleanline (Mullen, 2012), bad channel rejection (using channel power spectrum outlier thresholds), wavelet-thresholding of independent components, and secondary independent component analysis with component rejection via the automated multiple artifact rejection algorithm (MARA; Winkler et al., 2011, 2014). Data were then re-referenced to average, and segmented into 2-s windows for PAC analysis. This length was set to contain a sufficient sample of cycles for analysis of slower oscillations, while also retaining a sufficient amount of data (a longer segment length, in the presence of artifact, would lead to the rejection of more data). For each segment, to further remove artifact, the probability of an electrode’s activity given the electrode’s activity in all other segments, as well as the probability of an electrode’s activity given the activity of all other electrodes in the same segment, was assessed using EEGLAB’s pop_jointprob function (Delorme and Makeig, 2004). Segments where either probability was outside 3 SDs from the mean were rejected (Gabard-Durnam et al., 2018). Because 60 s of data has been shown to be needed to obtain stable PAC estimates (Berman et al., 2015), for each participant, 30 segments (60 s of data) were then randomly selected for further analysis; files with fewer than 30 segments of data at this stage were not analyzed (Table 2). Primary PAC metrics were then obtained as described below using code added to the BEAPP software, publicly available at https://github.com/lcnbeapp/beapp.

**Table 2:** Number of EEG files (for each net type and in total) collected and analyzed per age group studied

Age (months)	64-channel geodesic collected	64-channel geodesic analyzed	128-channel HydroCel collected	128-channel HydroCel analyzed	Total EEGs collected	Total EEGs analyzed
3	5	2	13	12	18	14
6	19	14	56	48	75	62
9	21	14	65	54	86	68
12	18	16	67	53	85	69
18	9	6	56	46	65	52
24	10	6	58	53	68	59
36	0	0	76	62	76	62

Five EEG recordings were >3 SDs from the mean on one of the following HAPPE data quality output parameters: percent good channels, mean retained artifact probability, median retained artifact probability, percent of independent components rejected, and percent variance retained after artifact removal. These EEGs were evaluated for differences in overall PAC (averaged across frequencies and channels), all files were found to be within 2 SDs of the mean at the time point of collection, so these files were included in later analyses.

#### Simulated signals

Our EEG processing pipeline included multiple filtering steps. Given that filtering of EEG data has been shown to result in frequency dependent phase shifts (Yael et al., 2018), we ran simulated signals through our pipeline to examine whether these filtering steps could alter observed PAC phase preference. To do so, a high-frequency oscillation was added on top of a low-frequency oscillation such that the high-frequency oscillation demonstrated increased amplitude during −90°, 0°, 90°, and 180° phases in four separate simulated signals. Simulated pink noise was then added to this signal to prevent artifact rejection from rejecting this unrealistic data. Because these phase shifts are frequency dependent, we generated these simulated phase-preferences in a variety of low-frequency and high-frequency combinations: specifically, between all combinations of the low-frequency oscillations of 2, 4, 8, and 16 Hz, and the high-frequency oscillation 64 Hz. All simulated signals were run through all processing steps used in the present study with the exception of re-referencing, which removes the phase preference of this simulated data. After running PAC, the phase preference of each low-frequency phase and high-frequency amplitude combination was compared with its original phase preference.

### Computation of PAC metrics

#### Modulation index

PAC was first quantified using the Pactools toolbox (Dupré la Tour et al., 2017) in Python as integrated within BEAPP. In detail, the signal in each segment was first exported from MATLAB into Python and filtered into a range of frequency pairs, with center frequencies for the low frequencies ranging from 2 to 20 Hz in 2-Hz steps, and for the high frequencies ranging from 40–100 Hz in 4-Hz steps. The low-frequency signal was filtered with a 2-Hz bandwidth, and the high-frequency signal was filtered with a 20-Hz bandwidth. Filtering at this step consisted of a zero-phase cosine-based filter to extract the real component, and then a sine-based filter to extract the imaginary component, resulting in a complex-valued output signal. Subsequently, PAC was assessed between each low-frequency and high-frequency filtered signal. Given the relatively wide bandwidth used when filtering the high frequencies, and to account for the transition bands of the filters, we did not analyze PAC frequency pairs in which there was overlap between the frequency response of the low-frequency filter and that of the high-frequency filter. For all other remaining low-frequency and high-frequency combinations, the time series of phases of the low-frequency signal and the amplitude of the high-frequency signal were obtained. The phases of the low-frequency signal were then binned into 18 20° intervals (−180° to 180°), and the mean of the amplitude of the high-frequency signal (HF_amp_) occurring within each phase bin was calculated. After applying this procedure on each of the 30 segments, data were then imported back into MATLAB. There, HF_amp_ at each phase bin of the low-frequency signal (LFϕ) was then averaged together across the participant’s 30 segments before computing the raw modulation index (MI_raw_) as the Kullback–Leibler divergence from a uniform amplitude distribution (see Tort et al., 2010 for more details and illustration). To control for factors not of interest that have been shown to affect PAC (such as spectral power), for each participant, 200 surrogate MI values (MI_surr_) were generated by repeating the procedure after offsetting HF_amp_ from LFϕ by a randomized time shift between 0.1 and 1.9 s. From this distribution, the mean [μ(MI_surr_)] and SD were then calculated. A normalized MI (MI_norm_) was then computed as the *z* score of the MI_raw_ compared with the distribution of MI_surr_ values (Canolty et al., 2006). Consequently, for each EEG, a single MI_raw_, μ(MI_surr_), and MI_norm_ were obtained. For subsequent clustering analysis, MI_raw_ values were statistically compared with μ(MI_surr_) values, while MI_norm_ values were used to assess a correlation with age.

#### Phase max

The Tort method used here to quantify PAC captures the presence of coupling, regardless of where in the LFϕ the high-frequency signal demonstrates increased amplitude. However, PAC can result from an increase high-frequency amplitude anywhere in the low-frequency waveform. To capture this, for each frequency pair at each electrode, we determined phase preference by calculating the LFϕ bin where HF_amp_ was maximal (termed phase max; Port et al., 2019). Phase max values were averaged across EEG recordings (in each age group, or across all age groups) using the circ_mean function in the circular statistics toolbox (Berens, 2009).

### Statistical analysis

#### Identification of PAC+ clusters across all ages

For every electrode, we now had obtained a modulation index for each frequency pair. To identify statistically significant areas both across frequency pairs and across channels, we employed a cluster-based permutation method described below to identify clusters exhibiting significant PAC (PAC+). We clustered on MI using data from all useable EEGs, at every age (three months to three years) at which they were collected.

Whereas most clustering procedures involve comparing one condition to another (i.e., task vs baseline stimuli), our task analyzed resting EEG data and therefore had no analogous comparison condition (or baseline) against which to test. In its place, we used the mean modulation index across the surrogate values μ(MI_surr_). These data serve as an effective “control” set as it retains the characteristics of the signal (power, noise), while any coupling present in the actual signal should not be retained. Thus, we used clustering against μ(MI_surr_) to reveal clusters where the PAC metric of interest is present to a significant degree.

For our clustering procedure, we implemented a non-parametric method that closely followed that by (Maris and Oostenveld, 2007):

1. For every channel, between every filtered low-frequency signal and high-frequency signal, a *t* test was used to compare the MI_raw_ values across all files and the μ(MI_surr_) values across all files.
2. Data points where the null hypothesis was rejected (*p* < 0.05, two-sided *t* test) were selected.
3. Selected data points on the same channel adjacent to one another in terms of low frequency or high frequency were grouped together into clusters (MATLAB function bwlabel, connectivity = 4).
4. After subtracting the minimum *t* value needed to achieve *p* < 0.05 from all data points, cluster level statistics were computed by taking the sum of *t* values within each cluster.

Then, to compute which clusters were significant (i.e., unlikely to occur with that strength or size by chance, or to remove clusters that may be spurious), we implemented the following:

1. For half of the participants, selected randomly, the MI_raw_ and μ(MI_surr_) data were “flipped,” such that their μ(MI_surr_) data were treated as MI_raw_, and their MI_raw_ were treated as μ(MI_surr_).
2. Test statistics and cluster sizes were calculated in the same manner as used previously.
3. The previous steps were repeated 200 times, creating a distribution of cluster sizes.
4. Clusters <95th percentile of this distribution were removed from further analysis.

For the remaining analysis, only low-frequency, high-frequency, and channel combinations belonging to a cluster found to demonstrate significant PAC (PAC+) were included.

#### Development of PAC metrics with age

To investigate how our PAC metrics in these clusters were associated with age, a Pearson correlation was computed between age and MI_norm_ averaged in all analyzed low-frequency, high-frequency, and channel combinations.

To analyze how PAC phase was associated with age, for each EEG recording, the number of low-frequency, high-frequency, and channel combinations analyzed where phase max was found in each LFϕ bin was taken. These values were then divided by the total number of low-frequency, high-frequency, and channel combinations analyzed to obtain the proportion of phase max values found in each phase bin. Age was then correlated with these values in all 18 LFϕ bins, indicating which LFϕ ranges demonstrated increases or decreases in proportion of phase max values with age. Significant correlations (*p* < 0.05) were determined after Bonferroni correction for 18 tests. Because a study being analyzed in parallel observed frontal and posterior channels differed in phase preference (Mariscal et al., 2021), we additionally performed these correlations in frontal and posterior channels separately.

### Code accessibility

The code to process EEG data are publicly available under the BEAPP and HAPPE software licenses (BEAPP: https://github.com/lcnbeapp/beapp; HAPPE: https://github.com/lcnhappe/happe). Additional code used for calculation of metrics and statistical analysis is available upon reasonable request.

## Results

### Simulated signals

All simulated signals were run through all preprocessing steps (with the exception of re-referencing) and their PAC was subsequently analyzed. All simulated phase max values in each low-frequency phase and high-frequency amplitude combination tested demonstrated its maximum high-frequency amplitude at its approximate simulated phase preference, indicating phase preference was not altered by the filtering steps used during EEG processing or PAC analysis.

### Identification of significant MI clusters across all ages

**Figure 2.**
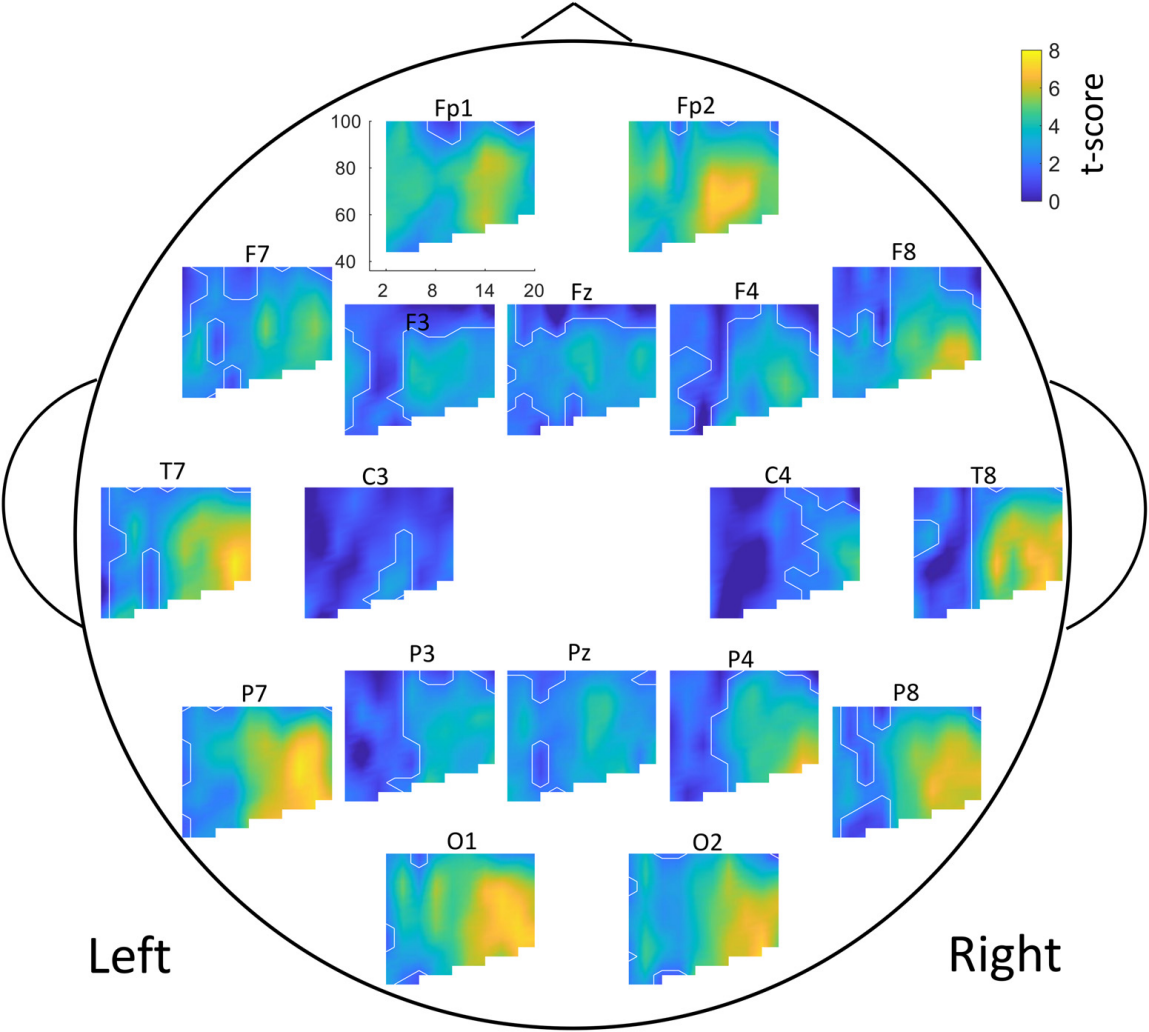
PAC+ clusters across all ages. Comodulograms of *t* scores, for each electrode, showing MI of each area. White lines outline clusters with significant MI (PAC+ clusters, *p* < 0.05, corrected for multiple comparisons). In some cases (e.g., Fp1, Fp2, O1, and O2), PAC+ clusters cover most of the comodulogram; therefore, in these cases, white lines outlining blue clusters mark the border of a small area that is not PAC+. Comodulograms indicate the level of coupling between phase frequencies (*x*-axis, 0–20 Hz), and amplitude frequencies (*y*-axis, 40–100 Hz). Each electrode is plotted at approximate electrode location on scalp. All analyzed EEGs were included (regardless of age at collection).

Clustering on MI of all participants regardless of age selected 68.2% of low-frequency, high-frequency, and channel combinations (Fig. 2). All channels analyzed contained at least one significant cluster.

### Phase preference

**Figure 3.**
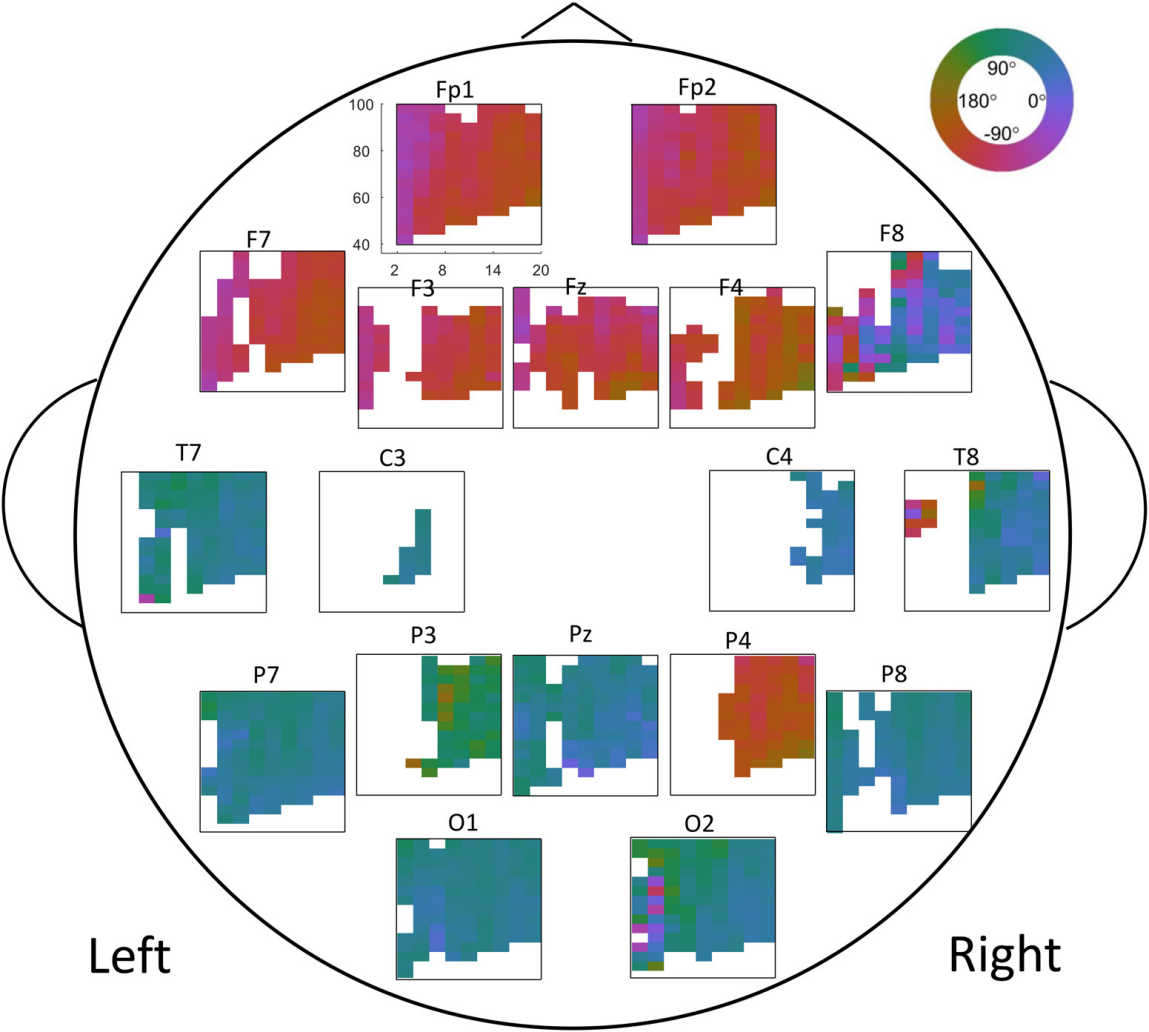
Phase preference across all ages. Comodulograms of phase max, for each electrode. Comodulograms indicate the average phase max value across all EEGs (regardless of age at collection). Here, only low-frequency, high-frequency, and channel combinations in PAC+ clusters are displayed. Each electrode is plotted at approximate electrode location on scalp.

**Figure 4.**
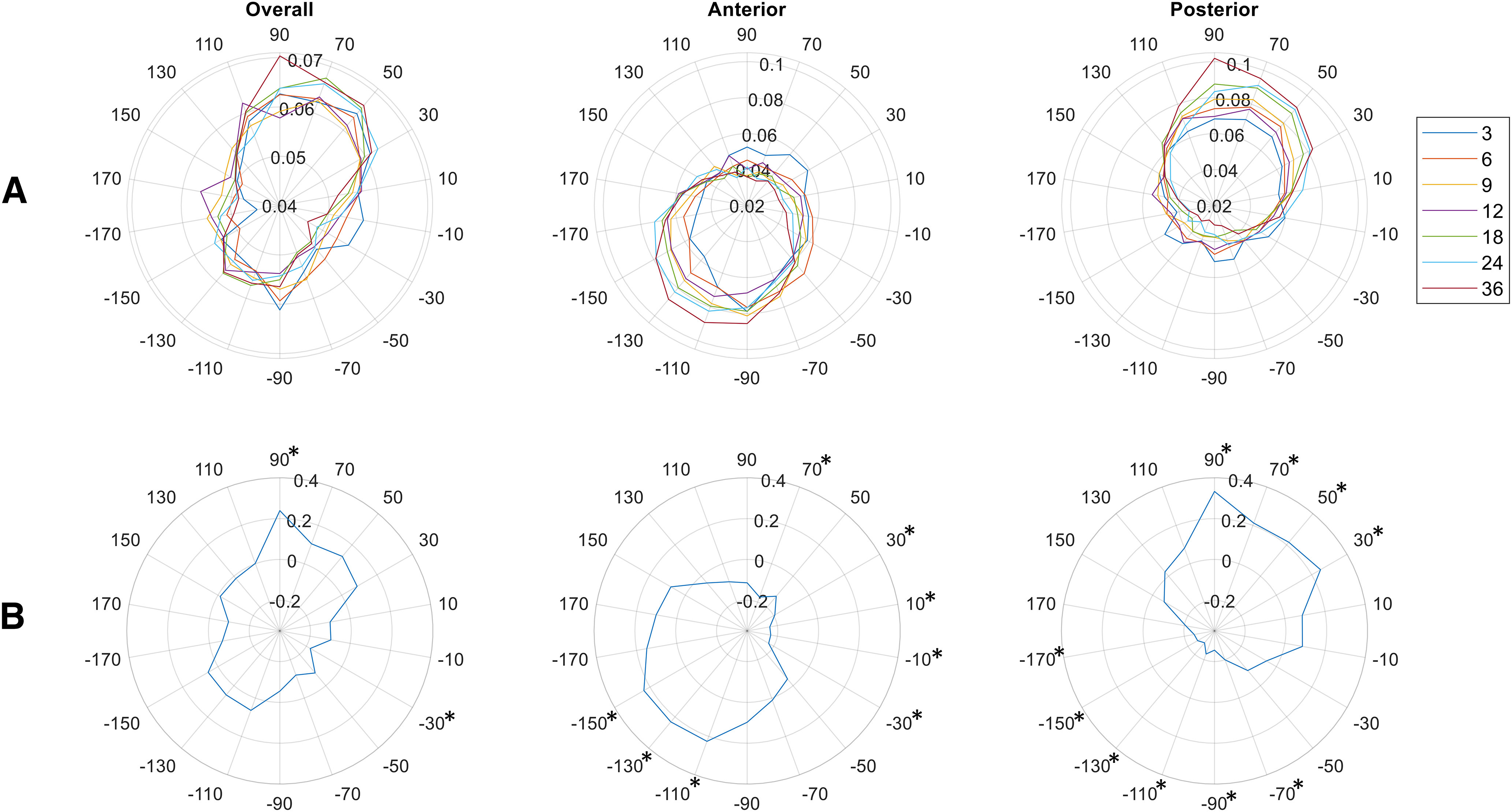
Distribution of phase max across all ages and correlations with age. ***A***, Circular plots display the mean proportion of low-frequency, high-frequency, and electrode combinations demonstrating phase max (radius of figure) at each phase bin (angles of figure) for each age (in months). Data are analyzed and plotted over all channels analyzed (left), anterior channels Fp1, Fp2, F3, F4, F7, F8, Fz (middle), and posterior channels P3, P4, P7, P8, Pz, O1, O2 (right). ***B***, Correlation coefficients (ρ, radius of figure) of the Pearson correlation between the proportion of low-frequency, high-frequency, and electrode combinations demonstrating phase max at each phase bin (angles of figure), and age. *Indicates phase bins where the correlation is significant (*p* < 0.05, Bonferroni corrected for 18 tests).

There is a spatial distribution of phase max, where more anterior electrodes largely exhibited a phase max on or around −110°, while more posterior electrodes largely exhibited a phase max on or around 70° (Figs. 3, 4*A*).

### Development of PAC metrics with age

**Figure 5.**
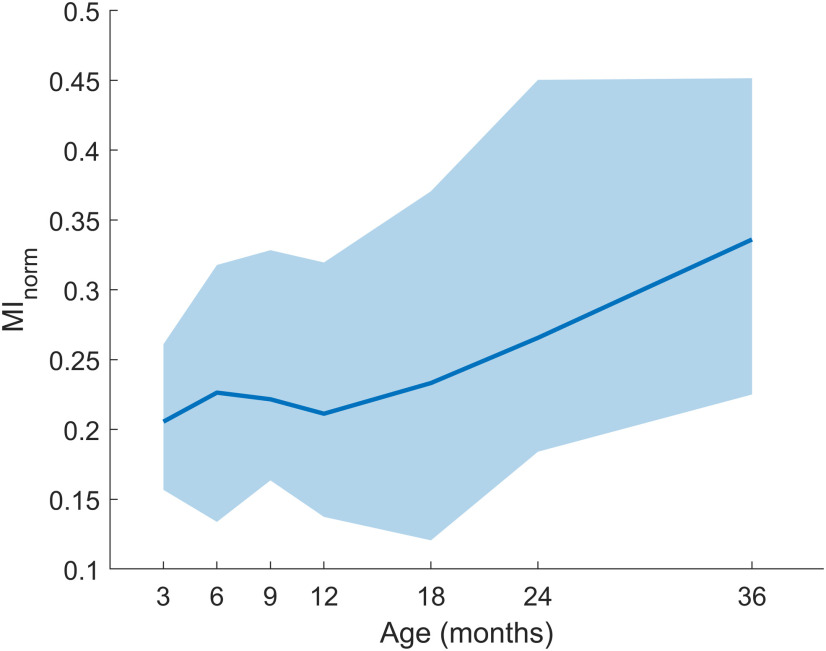
Association between MI_norm_ and age. Median MI_norm_ in PAC+ clusters plotted as a function of age. Clusters between the 25th and 75th percentiles are shaded.

MI_norm_ in PAC+ clusters increases with age (*r* = 0.2550, *p* = 2.035 × 10^−6^), this increase appears most prominently from one to three years of age (Fig. 5). MI_norm_ in PAC+ clusters does not differ as a function of net type (*p* > 0.1).

We additionally examined whether the proportion of low-frequency, high-frequency, and channel combinations demonstrating phase max at each phase bin is associated with age (Fig. 4). Over all channels analyzed, the proportion of data points demonstrating phase max in the 90° phase bin significantly increased with age, while the proportion of data points decreased in the −30° phase bin. This change was driven by the posterior channels, where the proportion of data points in the 30°–90° range increased with age, while the proportion of data points in the −170°–(−70°) range decreased. Anterior channels, on the other hand, demonstrated a nearly opposite trend, where the proportion of data points in the −150°–(−110°) range increased with age, while the proportion of data points in the −30°–70° phase range, with the exception of the 50° phase bin, decreased.

## Discussion

The primary goals in this study were to capture the dynamics of early brain development using PAC and to examine how PAC changes over the first three years of life. Here, we explore PAC from three months through three years of age. This PAC occurs most prominently between α-β and γ, largely consistent with several reports of α-γ PAC in resting state recordings (Roux et al., 2013; Berman et al., 2015; Gohel et al., 2016) but is also present between theta and γ. We observe PAC broadly across the scalp, suggesting a relatively ubiquitous presence of cross-frequency coupling. Our data suggest PAC increases in strength with age from three months to three years of age. The phase preference of this PAC shows opposing trends, separated by scalp region: PAC in posterior electrodes (particularly over occipital lobes) is driven by a peak in γ amplitude largely during the positive phases of LFϕ (centered around +70°), while PAC in anterior electrodes (particularly over frontal lobes) is driven by a peak in γ amplitude largely during the negative phases of the LFϕ [centered around −130°–(−110°)].

To our knowledge, this study is the first to demonstrate this regional separation in phase preference. As a result, the mechanisms underlying this difference, and its relationship to circuit function, is presently unclear. However, the strong difference of phase preference between brain areas, as well as the development of this difference with age, suggests phase preference may capture meaningful network characteristics. For example, laminar recordings have found phase preference to vary by cortical layers (Sotero et al., 2015). Because scalp-level EEG reflects the grand average of underlying activity, one possibility is that phase preference reflects the relative activity of underlying cortical layers; this would be of particular mechanistic interest because cortical layers with opposing phase preferences also have opposing predominant directionality of information flow [i.e., feedforward (bottom-up, thalamocortical) vs feedback (top-down, corticothalamic) activity]. Supporting this hypothesis, the relative thickness of each cortical layer differs along the anterior–posterior axis (Wagstyl et al., 2020). Back translation of phase preference findings, perhaps using laminar recordings, may provide further insights into neural network activity across development.

This study is not without limitations. Regarding the dataset analyzed, our sample size at the three-month time point was relatively small (*n* = 14), more participants would allow for a more exact description of PAC at this age. Likewise, most participants did not contribute quality data at every time point, meaning the sample composition at each age differs slightly from other ages. Because of this, our study demonstrates association between age and PAC at a group level, but does not directly demonstrate age-related changes on an individual level. In addition, although PAC has been shown to differ with vigilance state (He et al., 2010; Tokariev et al., 2016), this study only recorded from brief epochs of awake resting data, and consequently these effects were not analyzed. Likewise, our analysis was conducted on only 1 min of artifact-free data; although analytically, this recording length has been shown to be sufficient for PAC analyses (Berman et al., 2015), a longer recording would provide a more thorough description of PAC for each individual. Finally, the EEG analysis presented here was conducted on sensor (not source) level EEG data. This data could therefore be affected by changes in volume conduction because of the structural development of the brain during infancy (Grieve et al., 2003; Odabaee et al., 2014). Future studies are needed to assess how the structural changes in the brain during infancy could affect the results presented here.

In summary, this study documents the emergence of PAC in early childhood. We find HF_amp_ is increased at opposing phases of LFϕ in anterior areas as compared with posterior areas. Future studies would be beneficial in further assessing the potential functional relevance of the spatial distribution of phase preference; we suggest laminar differences in the direction of information flow (feedforward vs feedback) as one potential avenue for further exploration.

## Synthesis

Reviewing Editor: Niraj Desai, NINDS Decisions are customarily a result of the Reviewing Editor and the peer reviewers coming together and discussing their recommendations until a consensus is reached. When revisions are invited, a fact-based synthesis statement explaining their decision and outlining what is needed to prepare a revision will be listed below. The following reviewer(s) agreed to reveal their identity: Robert Knight. Note: If this manuscript was transferred from JNeurosci and a decision was made to accept the manuscript without peer review, a brief statement to this effect will instead be what is listed below.

The revision is impressive, and the paper is very interesting. One reviewer had a few (minor) additional comments for you to address.

**** FINAL REVIEW COMMENTS ****

Very nice revision. The question is interesting, the data are rich, and the findings are exciting. I have one minor remaining concern.

The authors clarified that the study followed a longitudinal design, but analyses did not explicitly model within-individual changes. This seems suboptimal given the data available but is acceptable. However, it is not okay to claim “change” is anything other than suggested if not shown explicitly. This should be rephased in the manuscript title and text.

Consider referencing Jones et al., 2020, NeuroImage, as evidence that PAC phase preference relates to task performance (intro, p. 3).

## Author Response

Re. Manuscript number eN-TNWR-0264-20X:

EEG Phase Amplitude Coupling and Phase Preference: Association with Age over the First

Three Years After Birth

Thank you for your consideration of our manuscript, and the opportunity to submit a revised

draft to eNeuro. One reviewer had a few (minor) additional comments for us to address. Our

point-by-point response to these comments is as follows.

Comment #1: “The authors clarified that the study followed a longitudinal design, but analyses

did not explicitly model within-individual changes. This seems suboptimal given the data

available but is acceptable. However, it is not okay to claim “change” is anything other than

suggested if not shown explicitly. This should be rephased in the manuscript title and text.”

Author's response: Thank you for this suggestion. In the manuscript title, we have removed the

term “Change.” The prior title was “Developmental Changes in EEG Phase Amplitude Coupling

Strength and Phase Preference Over the First Three Years After Birth,” and the new title is “EEG

Phase Amplitude Coupling Strength and Phase Preference: Association with Age Over the First

Three Years After Birth.” We have also made edits throughout the manuscript to our study

demonstrates association between age and PAC at a group level, but does not directly

demonstrate age-related changes on an individual level.

Comment #2: “Consider referencing Jones et al., 2020, NeuroImage, as evidence that PAC

phase preference relates to task performance (intro, p. 3).”

Author's response: Thank you for this suggestion. We have added this reference as

recommended
